# Association of *FCGR2A/FCGR3A* variant rs2099684 with Takayasu arteritis in the Han Chinese population

**DOI:** 10.18632/oncotarget.12738

**Published:** 2016-10-18

**Authors:** Si Chen, Xiaoting Wen, Jing Li, Yuan Li, Liubing Li, Xinping Tian, Hui Yuan, Fengchun Zhang, Yongzhe Li

**Affiliations:** ^1^ Department of Rheumatology and Clinical Immunology, Peking Union Medical College Hospital, Chinese Academy of Medical Sciences & Peking Union Medical College, Key Laboratory of Rheumatology and Clinical Immunology, Ministry of Education, Beijing, China; ^2^ Department of Clinical Laboratory, Beijing Anzhen Hospital, Capital Medical University, Beijing, China

**Keywords:** Takayasu arteritis, FCGR2A/FCGR3A, rs2099684, Chinese, Han

## Abstract

Takayasu arteritis (TA) is a chronic large-vessel vasculitis of unclear pathogenesis. A recent genome-wide association study (GWAS) has revealed that the *FCGR2A/FCGR3A, EEFSEC, RPS9/LILRB3, RIPPLY2* and *MLX* genes confer susceptibility to TA. We investigated the linkage between presumptive TA-related genes (*FCGR2A/FCGR3A, EEFSEC, RPS9/LILRB3, RIPPLY2* and *MLX*) and TA in the Han Chinese population.

We performed a large case-control multi-center study of 412 Han Chinese TA patients and 597 ethnically matched healthy controls. Five single nucleotide polymorphisms (SNPs) were assessed and genotyped using Sequenom MassArray system (iPLEX assay, Sequenom, San Diego, CA, USA).

The frequency of the rs2099684 variant G allele in the *FCGR2A/FCGR3A* gene was significantly higher in the TA patients than in the controls (37.5% compared with 25.4%, OR =1.77, 95% CI: 1.462.14, Pc =1.5×10^-8^). Similar results were observed in genotype distribution analysis and logistic regression analyses conducted using three genetic models. The allele and genotype distributions for the other polymorphisms were not significantly associated with TA among the Han Chinese patients.

The SNP rs2099684 in *FCGR2A/FCGR3A* can be considered a genetic risk factor for TA in the Chinese Han population. These findings provide further insights into the etiopathogenesis of TA.

## INTRODUCTION

Takayasu arteritis (TA) is a rare inflammatory disease typically characterized by non-specific inflammation of large arteries, especially the aorta and its major branches [1–3]. TA patients develop a wide range of symptoms, such as arterial stenosis, blood vessel wall thickening, dilation, progressive occlusion, and fibrosis, resulting in potentially life-threatening ischemia, aortic regurgitation, and pulselessness [1–3]. Other systemic symptoms may include myalgia, fatigue, arthralgia, fever, and weight loss. TA occurs all over the world and affects individuals of all ethnicities, but the highest prevalence rates have been described in Far East Asia, India, and Mexico, and this disease appears to be less common in European-derived individuals. Its reported incidence in North America is 2.6 cases per million per year [4]. However, its estimated incidence in Japan is approximately 40 cases per million [5]. Furthermore, the actual prevalence of this disease is unknown, as no large-scale epidemiological survey has been performed in China. TA is much more common in women, but the extent of this sex bias is dependent upon ethnicity and regional location [6, 7]. In addition, the average age of onset of this disease is between 20 and 40 years [6, 8].

The pathogenesis of TA has long been studied, but its etiology is largely unknown. The characteristic clinical features of TA, including the racial, ethnic, and geographic differences, indicate a potential role of genetic factors in its pathogenesis. In fact, several lines of evidence suggest a genetic contribution to TA considering the ethnic differences in its prevalence, familial aggregation, cellular autoimmunity and genetic factors, especially the repeatedly confirmed genetic association with the human leukocyte antigen (HLA) region across multiple ethnicities [9–16]. HLA-B*52 is the gene most significantly associated with TA in the *HLA* region among TA patients. Saruhan-Direskeneli et al. conducted a genome-wide association study (GWAS) of TA, in which they assessed Turkish and North American TA patients by dense genotyping and imputation analysis [17]. This group confirmed that the most significant genetic association was in the *HLA* region. In addition, they identified a genetic association in the region that includes the genes encoding Fc-gamma receptor IIA (*FCGR2A*) and Fc-gamma receptor IIIA (*FCGR3A*), as well as two additional association effects of proteasome (prosome, macropain) assembly chaperone 1 (*PSMG1*), interleukin-12 (*IL-12*) and IL-23 (*IL12B*) that conferred a risk of TA but did not pass the threshold for genome-wide significance. Moreover, associations with the same genetic variants in *IL12B* have been described and confirmed in a Japanese cohort of TA patients [18]. This group also discovered other non-HLA gene loci, including *EEFSEC* (rs10934853), *CCHCR1* (rs9263739), *RIPPLY2* (rs1570843) and *MLX* (rs665268) that conferred susceptibility to TA. Similarly, Renauer et al. carried out a GWAS on Turkish and North American TA patients and found that *IL6* (rs2069837), *RPS9/LILRB3* (rs11666543) and an intergenic locus on chromosome 21q22 (rs2836878) were associated with the disease [19].

It is widely accepted that racial and geographic differences and population heterogeneity are responsible for genetic predispositions to different diseases. The primary GWAS and candidate gene association studies were conducted on Turkish, North American and Japanese TA patients [17–19]. Thus, it is important to examine the relationships of the candidate genes with TA in the Han Chinese population. In the current study, we investigated gene polymorphisms in *FCGR2A/FCGR3A, EEFSEC, RPS9/LILRB3, RIPPLY2* and *MLX* in 412 Han Chinese TA patients and 597 ethnically matched healthy individuals to evaluate the genetic factors associated with TA in this population.

## RESULTS

### The subjects’ characteristics

A total of 412 adult-onset TA patients (85.0% women; mean age, 31.6±10.8 years) and 597 ethnically matched healthy controls (85.3% women; mean age, 36.2±10.2 years) were included in this study. The characteristics of the patients and controls are summarized in Table 1. All SNPs were in HWE in the healthy controls (P_HWE_>0.05). The genotype call rates of all SNPs were greater than 98%. The primary information for the five genotyped SNPs is shown in Table 2.

**Table 1:** Clinical data for TA patients and controls

Characteristic	Case	Control
Number of subjects	412	597
Female ratio (%)	85.0	85.3
Average age	31.6±10.8	36.2±10.2
Age at onset	27.2±9.6	NA
Onset age<20 years	85(20.6%)	NA
Hypertension	94(22.8%)	NA
Coronary artery involvement	11(2.7%)	NA
Ischemic brain disease	67(16.3%)	NA
Renal artery stenosis	111(26.9%)	NA
Pulmonary artery involvement	50(12.1%)	NA
Aortic regurgitation*	55(23.9%)	NA

* Only patients of Beijing Union Medical College Hospital were analyzed on aortic regurgitation for detailed clinical data available.

**Table 2:** Primary information for these SNPs

Genotyped SNPs	rs2099684	rs10934853	rs11666543	rs1570843	rs665268
Gene	*FCGR2A/FCGR3A*	*EEFSEC*	*RPS9/LILRB3*	*RIPPLY2*	*MLX*
Polymorphism	A>G	C>A	G>A	T>C	G>A
Chromosome	1	3	19	6	17
Function	Intergenic	intron region	nearGene-3	intergenic	missense
Chr Pos (NCBI)	161530340	128319530	54208113	83810801	42570011
MAF for Chinese in database*	0.36	0.39	0.10	0.49	0.40
MAF in our controls (n=968)	0.25	0.45	0.11	0.48	0.39
*Pc* for HWE test in our controls	0.45	0.16	0.13	0.32	0.86
Genotyping method	Sequenom MassArray iPLEX	Sequenom MassArray iPLEX	Sequenom MassArray iPLEX	Sequenom MassArray iPLEX	Sequenom MassArray iPLEX
Genotyping value (%)	100.0	99.9	98.3	100.0	99.8

NCBI: National Center for Biotechnology Information; MAF: minor allele frequency; *: the data were from the International HapMap Project; HWE: Hardy-Weinberg equilibrium.

### SNP analysis of the TA patients and controls from the Han population

The genotype and allele frequency distributions of the five SNPs are summarized in Table 3. The frequency of the rs2099684 variant G allele was significantly higher in the TA patients than in the controls (37.5% compared with 25.4%, OR = 1.77, 95% CI: 1.46-2.14, Pc = 1.5×10^-8^, Table 3). Similarly, a significant difference in the genotypic distribution of rs2099684 was detected between the TA patients and controls (Pc = 4.9×10^-7^, Table 3). However, no significant association with TA was detected for any of the other SNPs among the patients (all, Pc >0.05; Table 3). The logistic regression analysis results are summarized in Table 3. Logistic regression analyses based on the genetic additive, dominant and recessive models yielded similar results, with stronger associations between rs2099684 and TA observed using the additive model (OR = 1.72, 95% CI: 1.43-2.07, Pc = 7.4×10^-8^) and dominant model (OR = 1.87, 95% CI: 1.46-2.40, Pc = 5.0×10^-6^). However, no significant differences were detected between the TA patients and healthy controls for the other SNPs using any of the three genetic models (all, Pc >0.05, Table 4).

**Table 3:** Allele and genotype distribution of the gene markers in TA patients and controls

Gene	SNPs	Groups	Allele (%)		OR (95%CI)	*P*	*Pc*	Genotype (%)			χ^2^	*P*	*Pc*
			G	A				GG	GA	AA			
*FCGR2A/FCGR3A*	rs2099684	TA	309(37.5)	515(62.5)	1.77(1.46-2.14)	3.0×10^-9^	1.5×10^-8^	67(16.3)	175(42.5)	170(41.2)	32.27	9.8×10^-8^	4.9×10^-7^
		Controls	303(25.4)	891(74.6)				42(7.0)	219(36.7)	336(56.3)			
			A	C				AA	CA	CC			
*EEFSEC*	rs10934853	TA	344(41.8)	478(58.2)	0.90(0.75-1.07)	0.22	NS	22(18.7)	190(46.3)	144(35.0)	4.57	0.10	0.50
		Controls	533(44.6)	661(55.4)				110(18.4)	313(52.4)	174(29.2)			
			A	G				AA	GA	GG			
*RPS9/LILRB3*	rs11666543	TA	67(8.3)	737(91.7)	0.74(0.55-1.00)	0.05	0.25	3(0.7)	61(15.2)	338(84.1)	NA*	0.08	0.40
		Controls	129(10.9)	1051(89.1)				3(0.5)	123(20.8)	464(78.7)			
			C	T				CC	TC	TT			
*RIPPLY2*	rs1570843	TA	394(47.8)	430(51.2)	1.02(0.86-1.22)	0.82	NS	98(23.8)	198(48.1)	116(28.1)	1.61	0.45	NS
		Controls	569(47.7)	625(52.3)				129(21.6)	311(52.1)	157(26.3)			
			G	A				GG	AG	AA			
*MLX*	rs665268	TA	304(37.1)	516(62.9)	0.90(0.75-1.08)	0.24	NS	62(15.1)	180(43.9)	168(41.0)	1.99	0.37	NS
		Controls	467(39.1)	727(60.9)				90(15.1)	287(48.1)	220(38.8)			

TA: takayasu arteritis; OR: odds ratio; CI: confidence interval; χ^2^: Chi-square test; Pc: *P* value corrected by Bonferroni method; NA: not available; NA*: the P value of genotypic analysis was calculated under the logistic regression analysis; NS: not significant.

**Table 4:** Analysis of the five SNPs based on three genetic models

Gene	SNPs	Additive model		Dominant model		Recessive model	
		*Pc*	OR (95%CI)	*Pc*	OR (95%CI)	*Pc*	OR (95%CI)
*FCGR2A/FCGR3A*	rs2099684	7.4×10^-8^	1.72(1.43-2.07)	5.0×10^-6^	1.87(1.46-2.40)	4.9×10^-5^	2.50(1.67-3.75)
*EEFSEC*	rs10934853	NS	0.89(0.75-1.07)	0.21	0.76(0.58-0.99)	NS	1.03(0.75-1.42)
*RPS9/LILRB3*	rs11666543	0.25	0.96(0.78-1.20)	0.17	0.70(0.51-0.97)	NS	1.39(0.28-6.94)
*RIPPLY2*	rs1570843	NS	1.02(0.86-1.22)	NS	0.92(0.70-1.22)	NS	1.16(0.86-1.55)
*MLX*	rs665268	NS	0.90(0.75-1.08)	0.59	0.82(0.63-1.05)	NS	0.98(0.69-1.39)

OR odds ratio; CI confidence interval; Pc: *P* value corrected by Bonferroni method; NS: not significant.

## DISCUSSION

In this large-scale hospital-based case-control study, we investigated the associations of *FCGR2A/FCGR3A* (rs2099684), *EEFSEC* (rs10934853), *RPS9/LILRB3* (rs11666543), *RIPPLY2* (rs1570843) and *MLX* (rs665268) polymorphisms with the risk of TA in 412 Han Chinese TA patients and 597 ethnically matched healthy controls. Several gene variants associated with TA have been identified, but the three previous GWASs have been conducted on Turkish, North American and Japanese TA patients [17–19]. Therefore, further comparative studies are warranted to examine subjects of different ethnicities. The results of this study demonstrated a significant association between the *FCGR2A/FCGR3A* (rs2099684) polymorphism and TA among the patients. This result is consistent with previous findings reported for Turkish and North American TA patients [17]. Notably, this is the first study demonstrating a significant association between the *FCGR2A/FCGR3A* (rs2099684) polymorphism and susceptibility to TA in the Chinese Han population.

The rs2099684 locus is located between the *FCGR2A* and *FCGR3A* gene regions on chromosome 1:161524048-161541759. The *FCGR2A* gene encodes one member of a family of immunoglobulin Fc receptors that are found on the surfaces of many immune response cells. The protein encoded by the *FCGR2A* gene is a cell surface receptor present on monocytes, macrophages, neutrophils, natural killer (NK) cells, and T- and B-lymphocytes, and it participates in diverse functions, such as phagocytosis of immune complexes and modulation of antibody production by B cells. In addition, it has important roles in the GPCR and phospholipase C pathways. The *FCGR3A* gene encodes a transmembrane receptor for the Fc portion of immunoglobulin G that is involved in the removal of antigen-antibody complexes from circulation, as well as other antibody-dependent responses. This receptor is expressed on activated monocytes/macrophages, NK cells, and a subset of T cells.

Previous studies have revealed that the prevalence of other inflammatory diseases in TA patients is high, suggesting that TA may be associated with immune system abnormalities activated by other inflammatory diseases, such as infection or inflammatory diseases of unknown origin [20]. *FCGR2A/FCGR3A* has been reported to be a susceptibility gene for other autoimmune diseases, such as systemic lupus erythematosus [21, 22], rheumatoid arthritis [23], multiple sclerosis [24], type 1 diabetes [23] and ulcerative colitis [25, 26]. In addition, a genetic association between the *FCGR2A/FCGR3A* polymorphism and giant cell arteritis (GCA) has been previously identified in a small cohort from Spain [27]. The findings of these studies indicate that these two large-vessel vasculitides might share common predisposing genes. Previously, GCA and TA have been considered distinct disorders based on their differing ages of onset and ethnic distributions. However, reports have claimed that these disorders have more similarities than differences, including similarities in several clinical features, angiographic findings, and the histopathological characteristics of arterial lesions [28, 29]. Thus, our results indicate that *FCGR2A/FCGR3A* might be a susceptibility gene for TA in patients from the Chinese Han population, suggesting that TA might share associated genetic loci with other autoimmune diseases.

The *EEFSEC* (rs10934853), *RPS9/LILRB3* (rs11666543), *RIPPLY2* (rs1570843) and *MLX* (rs665268) polymorphisms were not found to be significantly associated with TA among the Chinese patients in our study, in contrast with the findings of previous studies. These inconsistencies in the results among studies may be due to differences in these loci between CEU (Utah residents with ancestry from Northern and Western European sample), JPT (Japanese in Tokyo, Japan) and CHB (Han Chinese in Beijing, China) in terms of linkage disequilibrium structure and minor allele frequency, and they may also be attributed to heterogeneous patient populations with racial and geographical differences. Therefore, the genetics of TA may be more complicated than previously thought. Additional studies of subjects of different ethnicities might elucidate whether SNPs in the *EEFSEC, RPS9/LILRB3, RIPPLY* and *MLX* genes are associated with susceptibility to TA.

Because TA is a rare autoimmune disease, early candidate gene studies only included small numbers of patients to increase statistical power. In some early studies, patients of different ethnicities were even investigated together. To avoid this limitation, our study enrolled a larger number of TA patients from the Chinese Han population than the early candidate gene studies. Therefore, the present investigation had sufficient statistical power to examine moderate and even marginal associations. Remarkably, our study revealed a significant positive association between the *FCGR2A/FCGR3A* (rs2099684) polymorphism and TA among the Chinese Han patients. Therefore, this SNP may play a potential role in the pathogenesis of TA. Nevertheless, we did not assess the role of the *FCGR2A/FCGR3A* (rs2099684) genetic variant in the development of TA in these patients.

In summary, the results of this study have demonstrated a strong relationship between the *FCGR2A/FCGR3A* (rs2099684) polymorphism and TA in patients from the Chinese Han population. These findings have provided supportive evidence and contribute to current knowledge about the pathogenesis of TA. Further investigations are needed to explore the associations of *EEFSEC, RPS9/LILRB3, RIPPLY* and *MLX* with TA in individuals of different ethnicities and from different regions.

## MATERIALS AND METHODS

### Subjects

This study was designed as a multi-center study that included 412 subjects with TA, diagnosed according to the American College of Rheumatology (ACR) criteria [30]. These patients were enrolled from two different sources; between February 2013 and July 2015, 230 patients were enrolled from the Peking Union Medical College Hospital. In addition, as this study was supported by the Research Special Fund for Public Welfare Industry of Health, 182 patients were recruited through the cooperation of 23 centers in China. Further, 597 healthy unrelated age- and sex-matched controls without any history of chronic disease were recruited from the Peking Union Medical College Hospital during physical examination. All patients and healthy individuals from the Chinese Han population of North China provided informed consent, and this study was approved by the Ethics Committee of the Peking Union Medical College Hospital.

### Selection of single nucleotide polymorphisms (SNPs)

Based on the findings of previous GWASs of TA patients, five SNPs (rs2099684, rs10934853, rs11666543, rs1570843 and rs665268) in the *FCGR2A/FCGR3A, EEFSEC, RPS9/LILRB3, RIPPLY2* and *MLX* genes were selected for analysis in this study (Table 2).

### Genotyping

We collected 2-mL peripheral blood samples from the TA patients and healthy controls and extracted DNA from the peripheral white blood cells of each participant using kits purchased from Tiangen (Beijing, China), according to the manufacturer’s instructions. The genotyping of five SNPs was accomplished using a Sequenom MassArray system (Sequenom iPLEX assay, San Diego, CA, USA) according to the manufacturer’s guidelines.

The primers for polymerase chain reaction (PCR) and for locus-specific single-base extension were designed using MassArray Assay Design 4.0 (Sequenom). First, all of the DNA samples from the patients and controls were transferred to a 384-well plate. Second, after multiplex PCR amplification, the products were used for locus-specific single-base extension reactions. Third, the final products were subsequently desalted and transferred to a 384-element SpectroCHIP array (Sequenom). Fourth, allele detection was performed by matrix-assisted laser desorption/ionization-time-of-flight mass spectrometry (MALDI-TOF MS). Finally, the resultant mass spectrograms and genotype data were analyzed using MassArray Typer 4.0 software.

### Statistical analysis

Statistical analysis was conducted using PLINK v1.07 software (Shaun Purcell, Boston, MA, USA) [31]. Hardy-Weinberg equilibrium (HWE) was assessed using the Chi-square (*X*^2^) test for each SNP. Any SNPs that deviated from HWE (*P* < 0.05 in the control groups) were excluded from subsequent analysis. The genotype and allele distributions between the TA patients and healthy controls were evaluated using the *X*^2^ test. *P-*values (corrected for multiple comparisons using the Bonferroni method) of less than 0.05 were considered statistically significant, and the odds ratios (ORs) and 95% confidence intervals (95% CIs) of associations were calculated. The genotype frequencies were further assessed using logistic regression models (additive, dominant, and recessive models).
